# Hole Repairing Algorithm for 3D Point Cloud Model of Symmetrical Objects Grasped by the Manipulator

**DOI:** 10.3390/s21227558

**Published:** 2021-11-13

**Authors:** Linyan Cui, Guolong Zhang, Jinshen Wang

**Affiliations:** Image Processing Center, School of Astronautics, Beihang University, Beijing 102206, China; cuily@buaa.edu.cn (L.C.); guolongzhang@buaa.edu.cn (G.Z.)

**Keywords:** 3D point cloud, hole repair, surface reconstruction, symmetry

## Abstract

For the engineering application of manipulator grasping objects, mechanical arm occlusion and limited imaging angle produce various holes in the reconstructed 3D point clouds of objects. Acquiring a complete point cloud model of the grasped object plays a very important role in the subsequent task planning of the manipulator. This paper proposes a method with which to automatically detect and repair the holes in the 3D point cloud model of symmetrical objects grasped by the manipulator. With the established virtual camera coordinate system and boundary detection, repair and classification of holes, the closed boundaries for the nested holes were detected and classified into two kinds, which correspond to the mechanical claw holes caused by mechanical arm occlusion and the missing surface produced by limited imaging angle. These two kinds of holes were repaired based on surface reconstruction and object symmetry. Experiments on simulated and real point cloud models demonstrate that our approach outperforms the other state-of-the-art 3D point cloud hole repair algorithms.

## 1. Introduction

With the development of machine vision, robots can use “eyes” to obtain external information directly like human beings, which improves the intelligence and autonomy of robots. For example, the eye-to-hand camera can model the grasped object and the surrounding environment with 3D reconstruction algorithms. The complete 3D models of the grasped object are vital in the subsequent tasks, such as planning and controlling the action of the manipulator. 

**Figure 1:**
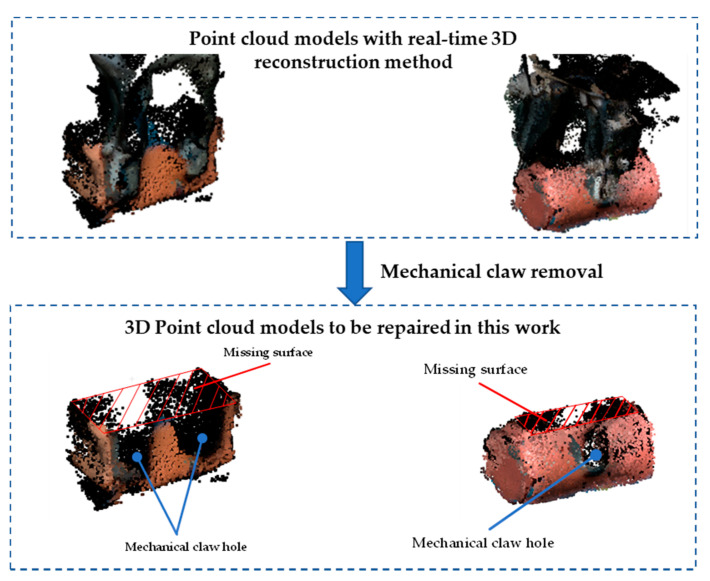
Examples of real-acquired 3D point cloud models repaired in this paper; the removal of the mechanical claw produces holes on the side of the point cloud; the limited imaging angle in 3D reconstruction results in a large missing surface at the top of the point cloud.

However, due to the occlusion of the mechanical claw and the limited imaging angle in 3D reconstruction, a large number of holes will appear in the point cloud model of the grasped object. Figure 1 provides examples of the real-time reconstructed point cloud models of symmetrical objects grasped by the manipulator with the ElasticFusion method [1]. The occlusion of the mechanical claw comes from the fact that the mechanical claw and the object are modeled together in the 3D reconstruction. After the mechanical claw is removed from the 3D point cloud model, several holes with a regular shape and medium size will be left on the side of the point cloud, whose shape is roughly equivalent to the contour of the mechanical claw. Due to the limited imaging angle in 3D reconstruction, the top of the object cannot be imaged when the manipulator grabs the object, resulting in a missing surface at the top of the point cloud. The missing surface can be regarded as a large area hole, and it exhibits different characteristics from the holes caused by the occlusion of the mechanical claw.

In our practical engineering, the 3D reconstruction and the mechanical claw removal are still in primary research; the high-quality point cloud models cannot be obtained for complex objects. Therefore, we limit the objects grasped by the manipulator to symmetrical objects, which can also provide more structural information for the hole repair. Based on the above application scenario, this paper mainly carries out the hole repair work on the 3D point cloud model of symmetrical objects grasped by the manipulator.

### 1.1. Related Work

The existing point cloud hole repair methods are mainly divided into two categories. The first includes traditional hole repair approaches, which detect and repair the holes by analyzing the characteristics of the point cloud itself and the hole distributions. Large amounts of data are not required. The second includes the approaches based on deep learning, which use the point cloud completion network to predict the missing area of the point cloud to repair the holes. A large amount of data are needed to train the network.

The traditional hole repair approaches can be further divided into methods based on the mesh model and those based on the scattered point cloud. The former methods use the connection topology information provided by the 3D mesh model to detect and repair the holes. For example, Chun-yen Chen et al. [2] proposed a 3D mesh model hole repair algorithm, which is based on a sharpness filter. Specifically, they adopted the interpolation method to fill the holes and utilized the regularized moving tetrahedron algorithm to reconstruct the missing surface. Z. Wei et al. [3] studied a hole-filling method based on surface fitting to repair the large missing surface. The mesh sub-division, optimization and weighed surface fitting were used to insert new sample points into the hole. Y.H. Wang et al. [4] utilized the triangulation method of base grid holes and the subdivision curved surface cutting out algorithm to obtain accurate filling data for all the holes. In summary, the above hole repair methods make use of a large amount of topological information between points provided by the mesh model; therefore, the hole repair effect is more accurate if the point cloud is clean and less noise exists. On the other hand, the usage of a large amount of topological information causes sensitivity to noise points and poor robustness. In addition, it requires high spatial complexity and prolonged computation time. 

Traditional hole repair methods that are based on the scattered point cloud directly process the scattered point cloud data that do not have a connection relationship between points. The holes are often filled by the geometric shape around them. Based on the physical diffusion model, L. Yang et al. [5] used two core steps of the normal propagation and the position sampling to recover the potential geometry shapes of the point cloud. H. Lin et al. [6] extracted the hole boundary and the point cloud feature lines for the holes with sharp features and divided them into feature and non-feature holes. Then, the authors used the tensor voting algorithm to fill all the holes; Y. Wang et al. [7] proposed a hole-filling method based on feature line extraction, which extracted a series of feature lines, such as the hole boundary lines, the ridge lines and the valley lines of the point cloud. Then, they filled the holes with the NURBS surface fitting method and retained the initial shape features of the holes. The hole repair algorithms based on the scattered point cloud model directly deal with the original point cloud data, so they spend less time repairing the holes. However, they do not effectively utilize the topology of the objects, and the hole repair accuracy needs to be further improved. 

Finally, the point cloud completion network based on deep learning focuses on inferring the possible shape of the point cloud from incomplete structures. The network is trained with a large amount of point cloud data and can complete the point clouds with different shapes and different missing conditions. For example, TopNet [8] generated the structured point cloud through soft constraints with the help of a flexible decoder, so as to avoid assuming a specific topology or point cloud structure on the underlying point set. With a sufficient training set, the model can theoretically learn the topological relationship on the point set. The PF-Net [9] estimated the missing point cloud hierarchically with the multi-scale generative network and added the multi-stage completion loss and adversarial loss, which can generate more real missing areas. The point cloud completion networks rely on a large amount of point cloud data. However, the existing 3D point cloud datasets are very different from the point cloud of objects grasped by the manipulator. For example, the ShapeNet dataset [10,11] used by PF-Net is too idealistic and sparse and cannot simulate the holes and noise in the point cloud of objects grasped by the manipulator. 

### 1.2. Motivation

The traditional hole repair methods based on the mesh model require high spatial complexity and prolonged computation time. The point cloud completion networks based on deep learning involve extensive collections of data to train the network. For the hole repair of the 3D point cloud of symmetrical objects grasped by the manipulator, the grasped objects have different shapes and variable sizes. In addition, in the real scene, due to the different grasping angles and depths of the mechanical arm, as well as different placement directions of objects, the holes in the point cloud model will appear in different spatial positions and have various distinct shapes. It is very difficult to prepare a hole repair dataset that covers a large number of point cloud models with different shapes, different grasping angles and different grasping depths. Furthermore, the noise and complex modeling conditions in the real scene make the real obtained point cloud models very different from the simulated ones. This limits the application of deep learning methods in this scenario.

In this work, traditional hole repair methods based on the scattered point cloud, which do not rely on a large amount of data for network training and have high robustness, were investigated for the hole repair of the 3D point cloud of objects grasped by the manipulator. We will take this as the basis to study our method and further improve the repair accuracy.

### 1.3. Contribution and Outline

In this paper, we propose a hole repair framework for the 3D point cloud of symmetrical objects grasped by the manipulator. This framework aims to increase the robustness and accuracy of the hole repair in variable scenes, such as the different grasping ways of the mechanical arm and the variable shapes and sizes of the grasped objects. 

Our contribution can be summarized as follows:

1. Design an automatic hole repair framework for the point cloud of symmetrical objects grasped by the manipulator, which can repair the mechanical claw holes and the missing surface automatically to obtain the complete point cloud.
2. Establish the virtual camera coordinate system to correct the pose of the 3D point cloud model and help calculate the bounding boxes to determine the filling regions of the holes.
3. Classify the hole boundaries automatically to deal with the nesting of the mechanical claw holes and the missing surface in the point cloud, and obtain their complete hole boundaries, respectively, which facilitates the different repair methods for the two kinds of holes.
4. Experiments on the simulated and real 3D point cloud models verify the hole repair effectiveness and robustness of the proposed framework.

The rest of the paper is structured as follows: Section 2 introduces the principle of the hole repair algorithm proposed in this work. The overall framework and the specific implementation methods are described in detail. Section 3 shows the hole repair effects on the simulated and real 3D point cloud models qualitatively and quantitatively. Finally, a summary is provided in Section 4.

## 2. Materials and Methods

### 2.1. Introduction to the Overall Framework

**Figure 2:**
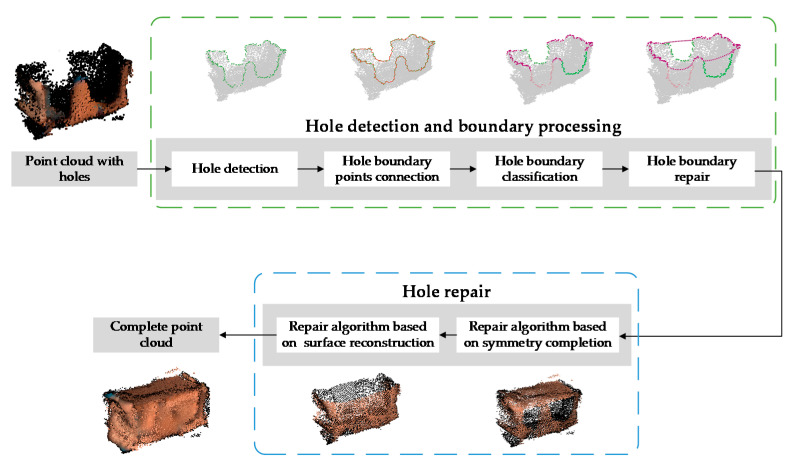
The flowchart of the proposed hole repair algorithm: the hole detection and boundary processing module and the hole repair module are included.

The flowchart of the proposed hole repair algorithm is shown in Figure 2. Two main modules, namely, the hole detection and boundary processing module and the hole repair module, are included. The former module consists of 4 steps. First, the method based on edge detection is used to detect the hole boundary points. Second, the discrete hole boundary points are clustered by connecting adjacent points and connected into lines to obtain the corresponding closed hole boundary of each hole. Third, in the case that multiple holes are nested in the boundary line, a boundary line classification method is proposed to distinguish the mechanical claw holes on the side of the point cloud and the missing surface at the top of the point cloud. Finally, the method of spline curve fitting and interpolation is used to repair the incomplete boundary lines after segmentation, and all the complete and closed hole boundaries of the point cloud are obtained.

Regarding the hole repair module, the hole repair algorithm is divided into 2 parts according to the characteristics of the mechanical claw hole and the missing surface in the point cloud. First, based on Poisson surface reconstruction, the surface of the object is estimated by fitting an indicator function determined by the point cloud, and the holes on the reconstructed surface are sampled randomly and evenly to repair the mechanical claw holes. Second, based on object symmetry, the missing surface at the top caused by the limited imaging angle is repaired with the intact area that is symmetrical to it.

### 2.2. Hole Detection and Boundary Processing

Before repairing the holes in the point cloud, first, the holes must be detected, and each hole should be described with complete and closed hole boundary lines. These hole boundary lines can provide the location and shape information of the holes, which will be used for the subsequent hole repair work. In this section, hole detection and boundary processing, which were carried out to acquire closed hole boundary lines, are described. 

#### 2.2.1. Hole-Detection Method Based on Edge Detection

The point clouds of symmetrical objects grasped by the manipulator studied in this paper were all scattered point clouds. There is no direct topological relationship between the points, so the edges of the point cloud are important geometric features to express the surface [12,13]. Additionally, the hole boundary is also a kind of edge of the point cloud. Therefore, we utilized the concept of edge detection as a reference to detect the holes.

Before detecting holes, the outlier points in the point cloud were removed with the Statistical Outlier Removal filter of PCL [14] to reduce the influence of noise on the detection accuracy.

A. Project the neighborhood points to the tangent plane.

First, we projected the neighborhood points onto a tangent plane, which helped us investigate the spatial distribution of neighborhood points more easily. 

For the point *P* to be investigated, *P* and its k-nearest neighbor points form the local reference point set. This point set is denoted as $\Omega =\{P_{i}(x_{i},y_{i},z_{i})|i=0,1,\cdots ,k\}$; $x_{i}$, $y_{i}$, $z_{i}$ are the spatial coordinates of point *P_i_*. According to the least squares method, the tangent plane of *Ω*, which is denoted as *T*, should satisfy the minimum sum of squares of the distance from the points in *Ω* to the plane *T*. The solution is as follows: let the plane equation of *T* be

(1) $$c_{1}x+c_{2}y+c_{3}z+c_{4}=0$$

then, it satisfies the matrix equation

(2) Ac=0

Among them:

$$A=\left[\begin{array}{cccc} x_{0} & y_{0} & z_{0} & 1\\ x_{1} & y_{1} & z_{1} & 1\\ \vdots & \vdots & \vdots & \vdots \\ x_{k} & y_{k} & z_{k} & 1 \end{array}\right],\;c={\left[\begin{array}{cccc} c_{1} & c_{2} & c_{3} & c_{4} \end{array}\right]}^{T}$$

The singular value decomposition method was used to solve Equation (2), and the eigenvector ***c*** corresponds to the maximum eigenvalue of $A^{T}A$. At this time, the tangent plane *T* was obtained. Additionally, the projection point set  Ω′ was obtained by projecting *Ω* to *T*. In  Ω′, the projection point of *P* was recorded as  P′, and the projection points of the neighbor points of *P* were recorded as ${\;Q\prime }_{i}\left(i=0,1,\cdots ,k-1\right)$.

**Figure 3:**
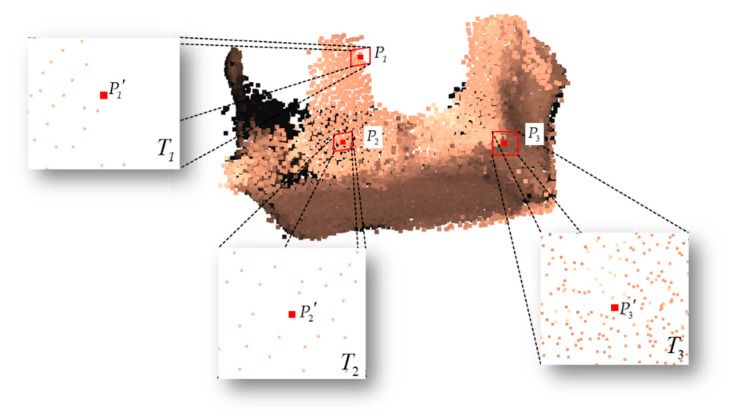
Example of projecting the neighborhood points to the tangent plane: $T_{1}$ , $T_{2}$ and $T_{3}$ are the tangent planes of the set of neighborhood points of $P_{1}$, $P_{2}$ and $P_{3}$, respectively.
$$P_{1}{}^{\prime }$$
, $P_{2}{}^{\prime }$ and $P_{3}{}^{\prime }$ are the projection points of $P_{1}$, $P_{2}$ and $P_{3}$, respectively.

The example of projecting the neighborhood points to the tangent plane is shown in Figure 3. Three points of $P_{1}$, $P_{2}$ and $P_{3}$ were chosen, which represent the hole boundary point, inner point and original edge point in the point cloud, respectively. $T_{1}$, $T_{2}$ and $T_{3}$ are the tangent planes of the set of neighborhood points, $P_{1}$, $P_{2}$ and $P_{3}$, respectively. $P_{1}{}^{\prime }$, $P_{2}{}^{\prime }$ and $P_{3}{}^{\prime }$ are the projection points of $P_{1}$, $P_{2}$ and $P_{3}$, respectively. The spatial distribution of the neighborhood points of these three points is displayed on the tangent plane intuitively.

B. Distinguish inner points, original edge points and hole boundary points.

As shown in Figure 3, $P_{1}$ is a hole boundary point, $P_{2}$ is an inner point and $P_{3}$ is an original edge point. After projecting the neighborhood points to the tangent plane, the neighbor points of $P_{2}{}^{\prime }$ are evenly distributed around it, while the neighbor points of $P_{1}{}^{\prime }$ are distributed on one side of $P_{1}{}^{\prime }$ unevenly. Additionally, as the surface of the point cloud of symmetrical objects grasped by the manipulator is closed and smooth, the neighbor points of $P_{3}{}^{\prime }$ are also evenly distributed around it, which is very similar to $P_{2}{}^{\prime }$. Therefore, the spatial distribution uniformity of neighborhood points was used to distinguish inner points, original edge points and hole boundary points.

**Figure 4:**
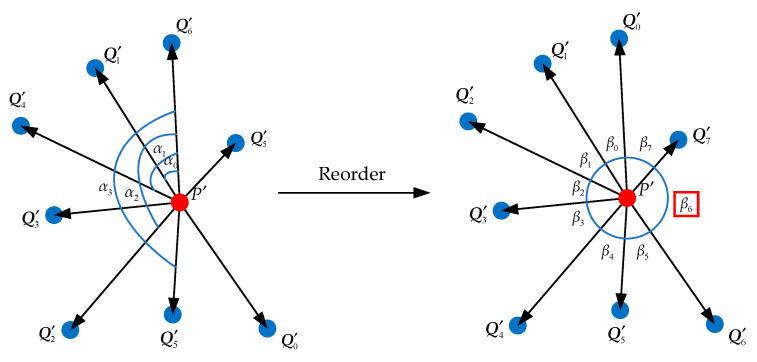
Maximum angle measurement criterion.

Then, the maximum angle measurement criterion was introduced to quantify the uniform distribution of the neighbor points. As shown in Figure 4, taking  P′ as the starting point and ${\;Q\prime }_{i}\left(i=0,1,\cdots ,k-1\right)$ as the ending points, a series of vectors $\vec{\;P\prime Q_{i}{}^{\prime }}\left(i=0,1,\cdots ,k-1\right)$ was obtained. Taking a vector $\vec{\;P\prime Q_{i}{}^{\prime }}$ randomly, first calculate the angle between it and other vectors $\vec{\;P\prime Q_{j}{}^{\prime }}\left(j\neq i\right)$, and this angle is denoted as $\alpha_{m}\left(m=0,1,\cdots ,k-2\right)$. Second, reorder the vectors according to the angle $\alpha_{m}$ along the counterclockwise direction. Third, calculate the angle between two adjacent vectors for the sorted vector group, which is denoted as $\beta_{i}(i=0,1,\cdots ,k-2)$. Take the maximum angle *β*. If *β* is greater than the angle threshold, the point *P* is judged as a hole boundary point. Otherwise, it is judged as an inner point or an original edge point.

In this work, the hole boundary points were detected accurately by setting the angle threshold to π/2 in the simulated and real point cloud models. However, we found that when the simulation model had very sharp edges (such as the triangular prism model in Section 3.2), using the fixed threshold of the maximum angle criterion could not robustly distinguish all the original sharp edge points from hole boundary points, although the method is applicable to most of the models. We suppress the influence of the possible errors caused by the very sharp edges of the point cloud in Section 2.2.2.

**Figure 5:**
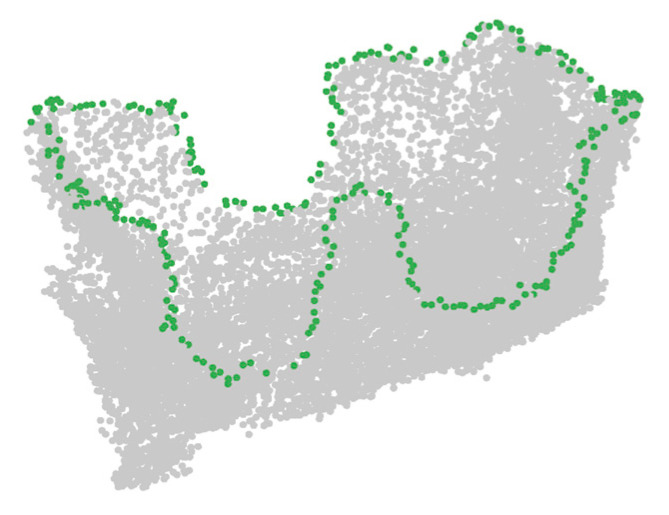
Example of hole boundary point detection result: the green points are the hole boundary points.

The hole boundary point detection effect is shown in Figure 5, and they are disordered points.

#### 2.2.2. Hole Boundary Point Connection Method Based on Neighbors

**Figure 6:**
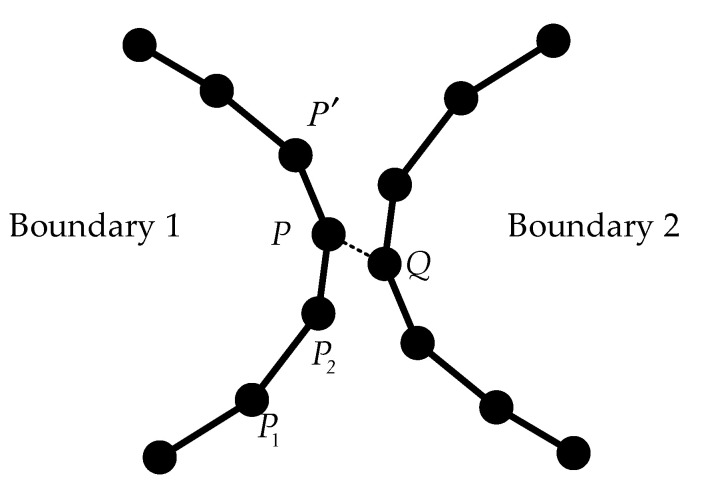
Two holes “adhere” to each other.

After detecting the hole boundary points, it is necessary to connect the disordered boundary points to a closed boundary line. As the boundary points belonging to the same hole are adjacent to each other in the 3D space, the KNN method (K-nearest neighbors) can be used to construct the boundary line; that is, a random hole boundary point is selected as the starting point, and its nearest boundary point is connected until the boundary line is closed. However, this method also has disadvantages. If 2 holes are too close to each other, only depending on the distance as a criterion will cause the boundary points of the 2 holes to “adhere” to each other. Therefore, the connection direction was taken as another criterion. The points of a single boundary line were connected one by one. If the new point caused the angle of the boundary line to deflect obviously, it was treated as a point of other boundary lines. Specifically, as shown in Figure 6, for a certain boundary point $P_{2}$ that was considered to belong to Boundary Line 1, its previous point in line 1 is $P_{1}$, and its nearest neighbor point is *P* and *Q*. Connecting each point only by distance is likely to result in *Q* as the next connection point, resulting in a connection error. Therefore, for each candidate connection point, the vector deflection angle [15] was calculated as the criterion of the connection direction.

(3) $$\theta (P)=\arccos(\frac{\vec{P_{1}P_{2}}\cdot \vec{P_{2}P}}{\left|\vec{P_{1}P_{2}}\right|*\left|\vec{P_{2}P}\right|})$$

The point with the smallest *θ* is regarded as the final connection point. Since θ(P)<θ(Q), *P* was selected as the next connection point of Boundary 1 after $P_{2}$.

**Figure 7:**
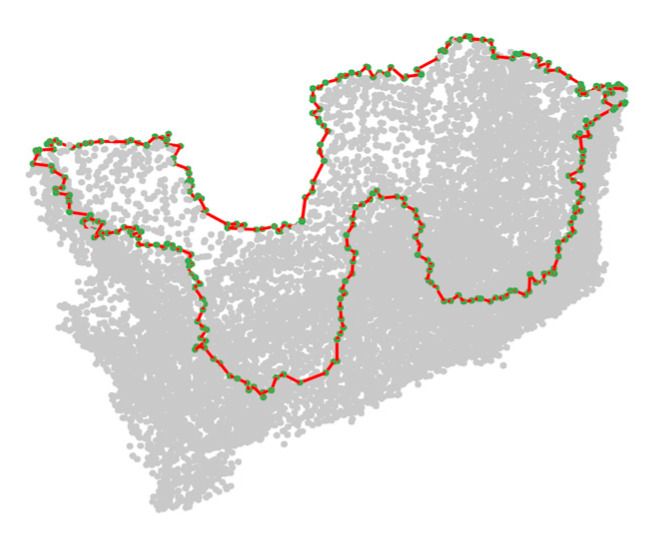
Example of hole boundary point connection.

By connecting hole boundaries, we can delete the unclosed and isolated hole boundaries, including the incorrectly detected hole boundaries caused by the very sharp edges of the point cloud, which ensures that they will not affect the subsequent hole repair. An example of a hole boundary point connection result is shown in Figure 7. It can be seen that the boundary points are connected in an ordered line. However, this algorithm cannot distinguish the boundary lines of different holes. The closed boundary of each hole cannot be obtained, which is due to the fact that the holes in the point cloud are nested, and the boundaries of different holes intersect. We solve this problem in Section 2.2.3.

#### 2.2.3. Hole Boundary Classification Method

**Figure 8:**
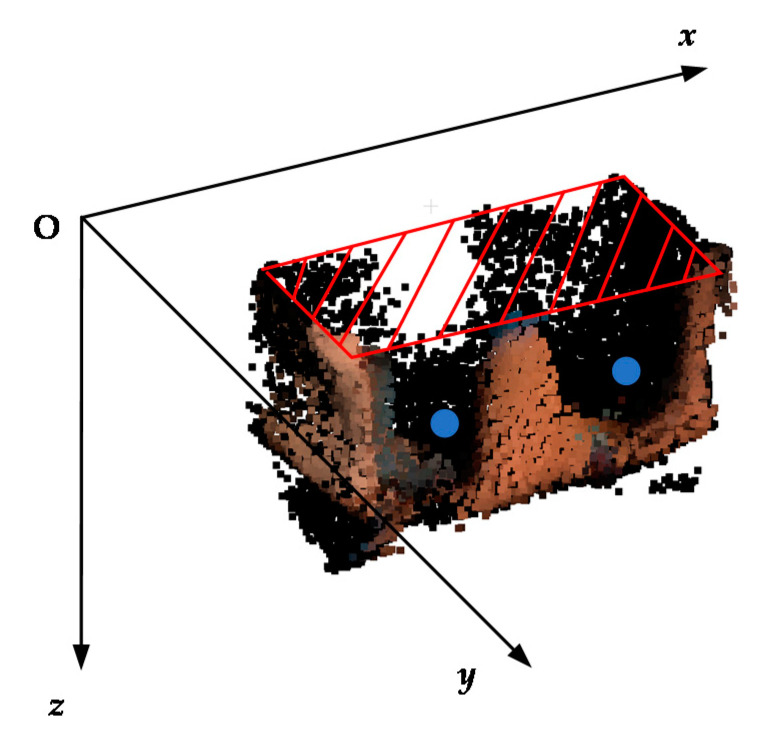
The virtual camera coordinate system.

Due to the fixed camera angle, the missing surface on the point cloud will always appear at the top of the camera coordinate system, and for the object grasped in practical engineering, the straight up and down grasping method will often make the bottom of the object perpendicular to the main direction of the mechanical claw. Therefore, the relative position between the missing surface and the mechanical claw hole can be used to distinguish these 2 kinds of holes. However, the pose of the object point cloud model is often random, and the real camera coordinate system information is commonly not given. Therefore, we established a virtual camera coordinate system to correct the pose of the point cloud model (as shown in Figure 8). Then, the *z*-axis coordinate was used to distinguish the relative position between the missing surface at the top of the point cloud and the mechanical claw hole on the side of the point cloud. 

Next, the details for establishing the virtual camera coordinate system and classifying the hole boundary line are provided.

A. Establish virtual camera coordinate system.

First, we needed to determine the plane of the missing surface boundary. The fitting plane for hole boundary point set of point cloud was acquired by the RANSAC algorithm [16,17], and we obtained several fitting planes (denoted as $\gamma_{1},\gamma_{2},\cdots \gamma_{m}$) and their normal vectors (denoted as ${\vec{n}}_{1},{\vec{n}}_{2},\cdots {\vec{n}}_{m}$). They are the fitting planes for the bottom or the side of the point cloud. We used the knowledge that the plane of the mechanical claw hole boundary and the plane of the missing surface boundary are orthogonal to each other to distinguish them. Thus, the sum of the dot product of each normal vector and other normal vectors was calculated as follows:

(4) $$\mathrm{dots}(\gamma_{k})=\sum_{i\neq k}^{m}{\vec{n}}_{k}\cdot {\vec{n}}_{i},(k=0,1,\cdots ,m)$$

The fitting plane with the smallest dots value was regarded as the plane of the missing surface boundary, and it was taken as the horizontal plane of the virtual camera coordinate system. The direction orthogonal to the horizontal plane was taken as the *z*-axis. Then, we projected the point cloud to the horizontal plane, drew the 2D bounding box of the projected point cloud and took the edges of the 2D bounding box as the *x*-axis and *y*-axis. The virtual camera coordinate system was established as shown in Figure 8. Finally, we projected the point cloud in the virtual camera coordinates.

B. Hole boundary classification based on the value in the *z*-axis.

**Figure 9:**
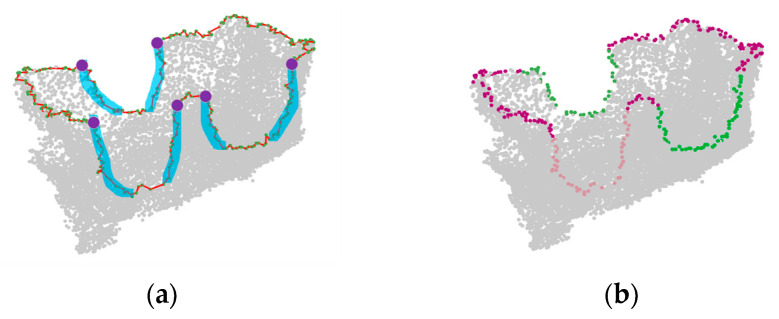
Example of hole boundary classification: (**a**) the segments with a continuous height increase or decrease (marked in blue) and the demarcation points (marked in purple); (**b**) hole boundary classification result; the boundary of the missing surface is marked in dark red; the boundaries of the mechanical claw holes are marked in green, light green and pink.

Through the above projection, the value in the *z*-axis of the boundary points can be used to determine whether the point belongs to the missing surface boundary or the mechanical claw hole boundary. The boundary lines in Section 2.2.2 are connected in order, and the subsequent steps of repairing the hole boundary still rely on this. Starting from the boundary point with the smallest *z*-axis coordinate (which belongs to the missing surface boundary), we traversed each boundary point in the order of the boundary lines in Section 2.2.2 to find all the segments with a continuous height increase or decrease (as shown in Figure 9a). Additionally, in order to reduce the noise interference, these segments should contain more than 5 points. The starting point and the ending point in these segments are the demarcation points of the missing surface boundary and the mechanical claw hole boundary, respectively. Then, we can classify these 2 kinds of holes with these demarcation points.

The classification result of the hole boundary line is shown in Figure 9b. It can be seen that the missing surface and the 3 mechanical claw holes are classified correctly.

#### 2.2.4. Hole Boundary Repair Method Based on Spline Curve Fitting and Interpolation

After the hole boundary line classification in Section 2.2.3, several unclosed boundary lines were generated, including the missing surface boundary and the mechanical claw hole boundaries. They all have different degrees of deficiency, which need to be repaired for complete and closed hole boundaries. 

**Figure 10:**
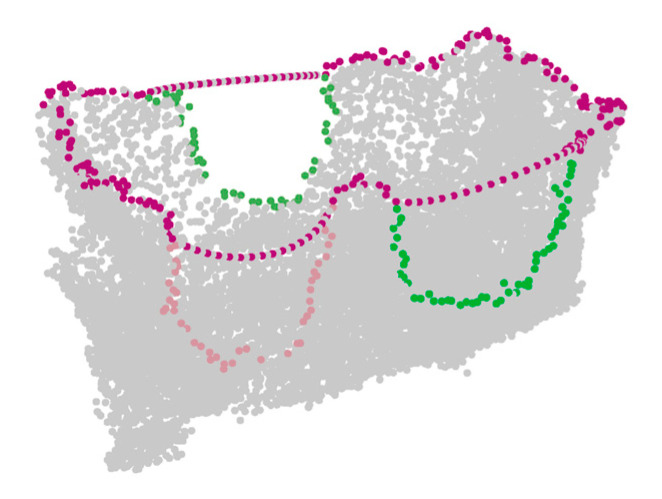
Example of the hole boundaries repair result.

All the points of the missing surface boundary were used as the control points to approximate the cubic B-spline curve, and the estimated curve equation was obtained. Then, the missing surface boundary was interpolated uniformly according to the estimated curve equation. The interpolation points obtained above were also used to repair the mechanical claw hole boundaries. Through this step, the closed hole boundary of each hole was obtained, as shown in Figure 10. 

### 2.3. Hole Repair

After the hole detection and boundary processing, the complete and closed hole boundaries were obtained, including the missing surface boundary and the mechanical claw hole boundaries. Based on the position information of the holes provided by the hole boundaries, this section describes the hole repair method. Combined with the characteristics of these 2 kinds of holes, the mechanical claw hole repair method based on surface reconstruction and the missing surface repair method based on symmetry completion are proposed, respectively.

#### 2.3.1. Repair Algorithm of Mechanical Claw Holes Based on Surface Reconstruction

The holes caused by the mechanical claw occlusion have regular shapes and appear to be in a relatively fixed position. Additionally, the regions that appear on the side of the point cloud and have no holes have relatively complete surfaces. Based on the above analysis, we first used Poisson surface reconstruction [18,19,20,21] to fit the regions that appeared on the side of point cloud, and the closed surfaces with good details were obtained as the fitting plane of the mechanical claw holes. Specifically, we used all the points on the side of the point cloud as the input points, which was more likely to restore the possible shape of the claw hole since the side of the point cloud model maintained a good shape in the non-hole area. We implemented Poisson reconstruction based on PCL [22]. The maximum depth of the Octree was set to 6. The minimum number of sample points that should fall within an octree node was set to 16. The ratio between the diameter of the cube used for reconstruction and the diameter of the samples’ bounding cube was set to 4.0. Other parameters followed the default settings of PCL.

Then, we calculated the bounding boxes of the mechanical claw holes. Since the virtual camera coordinate system was established in Section 2.2.3, the fitting plane of the mechanical claw hole boundary was basically perpendicular to the xOy plane of the virtual camera coordinate system. The bounding box of each mechanical claw hole was determined according to the maximum and minimum coordinate values of the hole boundary points of the mechanical claw. This obtained bounding box could effectively describe the range and location of the mechanical claw hole. 

Finally, random uniform sampling was performed in the above-calculated bounding boxes. For each bounding box, the number of sampling points (denoted as *N*) was calculated according to the volume of this bounding box (denoted as *V*) and the average distance between every point in the bounding box and their nearest neighbors (denoted as $\bar{D}$).

(5) $$N=V/{\bar{D}}^{3}$$

We selected *N* random points within the bounding box, determined the points that conformed to the reconstructed surface equation and inserted them into the origin point cloud. 

**Figure 11:**
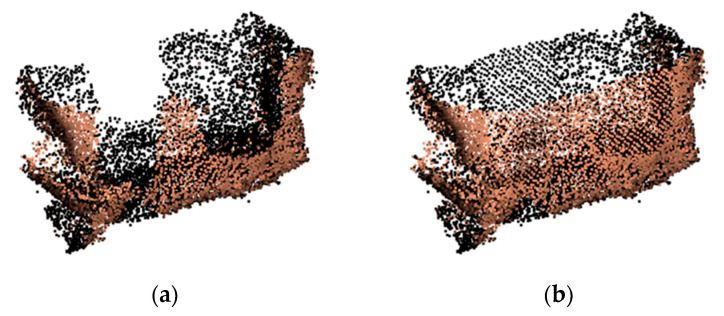
Example of mechanical claw hole repair result: (**a**) before repair; (**b**) after repair.

The repair effect for the holes caused by the mechanical claw occlusion is shown in Figure 11. It can be seen that the mechanical claw holes are repaired effectively.

#### 2.3.2. Repair Algorithm of Missing Surface Based on Symmetry Completion

Due to the limited imaging angle in 3D reconstruction, the missing surface often appears at the top of the point cloud. As the symmetrical objects grasped by the manipulator have different shapes, the shape of the missing surface is uncertain, and it cannot be predicted by the neighborhood points. At this time, the surface reconstruction method mentioned in Section 2.3.1 is no longer applicable. To solve the problem, this section proposes a missing surface repair algorithm based on the symmetry completion. For the symmetrical object in practical engineering, the missing surface must be perpendicular to the main direction of the mechanical claw, so the missing surface at the top of the point cloud can be repaired by its symmetrical surface at the bottom of the point cloud. The specific steps are as follows.

**Figure 12:**
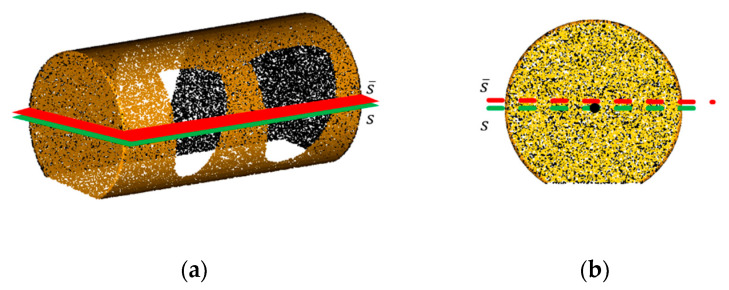
Example of the symmetry plane of point cloud: (**a**) cylinder point cloud with holes; (**b**) side view of point cloud.

In Section 2.2.3, the pose of the point cloud was corrected according to the established virtual camera coordinate system, so that the 3 axes of the point cloud were aligned with the 3 axes of the virtual camera coordinate system. In this case, the symmetrical surface of the missing surface was parallel to the horizontal plane of the virtual camera coordinate system. To repair the missing surface, first, we needed to find the maximum, minimum and median values in the *z*-axis of the point cloud. Then, at the median value position of *z*-axis, the cross section that was parallel to the xOy plane of the point cloud was acquired. By adjusting this cross section along the *z*-axis, the cross section with the maximize area was obtained and taken as the accurate symmetric plane of the point cloud model. As shown in Figure 12, in the cylinder point cloud model with a missing surface at the top (rotate to the bottom for the convenience of display), its symmetric plane determined by the median value in the *z*-axis directly (marked as $\bar{s}$) is higher than the real symmetric plane (marked as *s*) due to the existence of the missing surface. Then, combined with the boundary line of the missing surface obtained in Section 2.2.4, the bounding box for the missing surface was drawn to determine the filling area just as in Section 2.3.1, and all the points on the symmetric position of the bounding box were filled into the missing surface. Finally, we retained all the points of the original point cloud in the overlapped region, in order to preserve the structure information of the original point cloud and the non-hole region.

**Figure 13:**
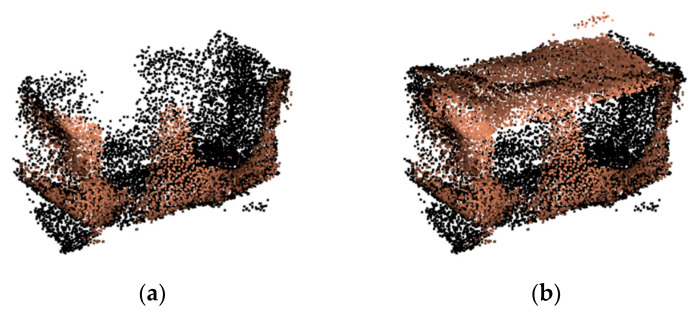
Example of missing surface repair effect: (**a**) before repair; (**b**) after repair.

The repair result for the missing surface in the point cloud is shown in Figure 13. There is a good repair effect for the missing surface at the top of the point cloud.

## 3. Results and Discussions 

In this section, experiments performed for both simulated and real acquired 3D point cloud models to verify the effectiveness and robustness of the hole repair algorithms proposed in this paper are described. 

### 3.1. Evaluation of Hole Repair Effect for Simulated 3D Point Cloud Model

The simulated point cloud models were produced with the SolidWorks software. To simulate the holes in the point cloud model caused by the mechanical claw occlusion and limited imaging angle in 3D reconstruction, the top region of the simulated point cloud model was cut off, and three holes with the size of the mechanical claw were dug out on the side of the point cloud model. Simulated point cloud models cover many symmetrical shapes, such as a cuboid, triangular prism, cylinder, sphere and elliptic cylinder. 

As the simulated models had the ground truth, the chamfer distance was introduced as the quantitative evaluation index:

(6) $$d_{CD}(S_{1},S_{2})=\frac{1}{S_{1}}\sum_{x\in S_{1}}\underset{y\in S_{2}}{\min}{\left\|x-y\right\|}_{2}^{2}+\frac{1}{S_{2}}\sum_{y\in S_{2}}\underset{x\in S_{1}}{\min}{\left\|y-x\right\|}_{2}^{2}$$

where $S_{1}$ represents the point cloud model that must be evaluated, and $S_{2}$ is the ground truth that has no holes. The first term on the right side of the equation denotes the average of the minimum distance from any point *x* (in $S_{1}$) to $S_{2}$, and the second term represents the average of the minimum distance from any point *y* (in $S_{2}$) to $S_{1}$. The chamfer distance was used to measure the difference between the two point clouds. The smaller the $d_{CD}$ value is, the smaller the difference between $S_{1}$ and $S_{2}$ is.

#### 3.1.1. Comparative Experiments among Different Algorithms

**Figure 14:**
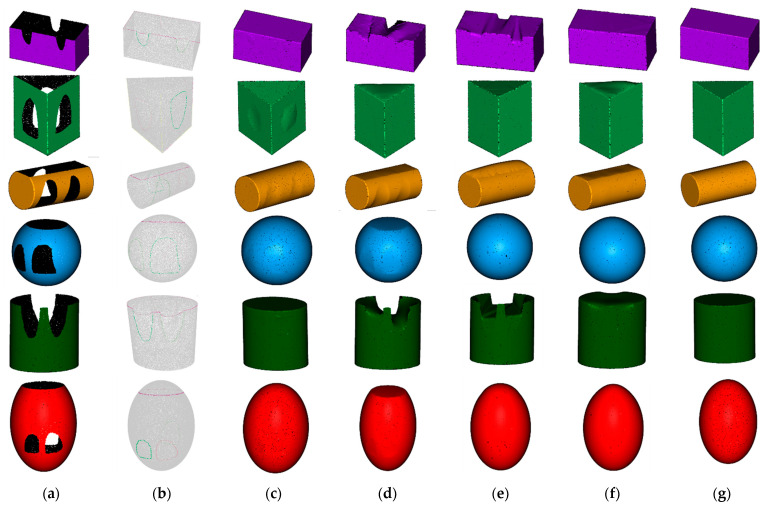
Repair effect of simulated point cloud model: (**a**) before repair; (**b**) hole detection result with our algorithm; (**c**) ours; (**d**) DC-AB scanner; (**e**) Meshmixer; (**f**) Geomagic; (**g**) ground truth.

While verifying the hole repair effect of the algorithm proposed in this paper, the hole repair algorithms used by the DiChuang DC-AB 3D scanner [22], the Meshmixer software of Autodesk company [23] and the Geomagic Studio software of Geomagic company [24] were also introduced for comparison. The hole repair effects of the simulated point cloud models are shown in Figure 14. It can be seen that our algorithm achieved the best hole repair effects for most of the point cloud models. The hole-filling algorithms used by the DC-AB 3D scanner and the Meshmixer software exhibited slightly better repair effects for the mechanical claw holes, such as the triangular prism and the cylinder. However, they were unable to effectively repair the missing surface in the point cloud, and the overall repair effects for the point cloud were poor, as shown in Figure 14. The hole-filling algorithm used by the Geomagic Studio software performed better than the former two algorithms in the overall hole repair effect, but there were also some errors in the cylinder model, as the side of the cylinder was repaired to a plane. In terms of hole repair speed, our algorithm took approximately 1 s, which is almost the same as that of the other three algorithms.

In addition to the visual comparisons, the chamfer distance was calculated and is listed in Table 1. It can be seen that the chamfer distance is significantly reduced with our algorithm. Compared with other algorithms, except for the triangular prism model and the cylinder model (in the second and third rows), our hole repair algorithm achieved the minimum chamfer distance, which is consistent with the visual results shown in Figure 14. The reason that our algorithm achieved a slightly poorer hole repair effect for the triangular prism model and the cylinder model is that the mechanical claw holes occupy a large proportion of the whole point cloud model, which affects the quality of Poisson reconstruction. 

#### 3.1.2. Comparative Experiments among Models of Different Sizes

**Figure 15:**
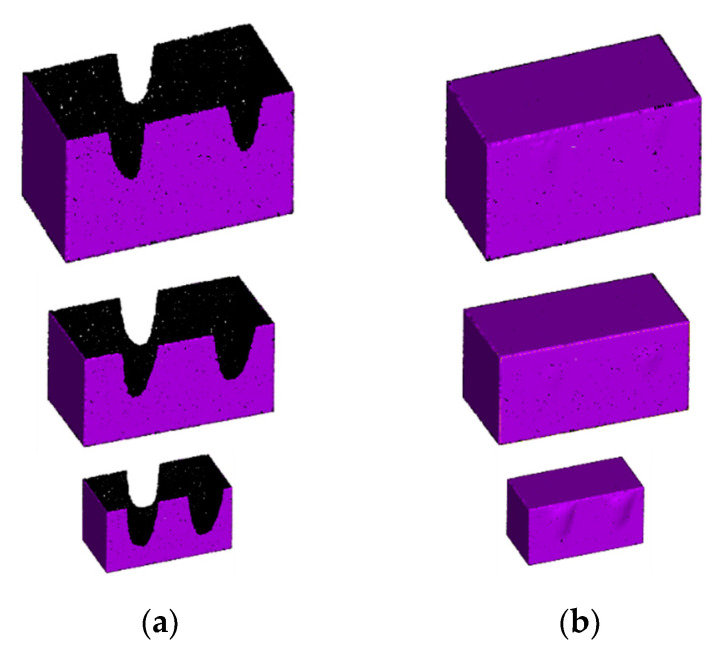
Repair results of cuboid point cloud models with different sizes: (**a**) before repair; (**b**) after repair.

In the real application scene, the mechanical arm often needs to grasp objects with different sizes. To investigate the robustness of our method on the point cloud models with variable sizes, cuboid point cloud models with different sizes were simulated as examples. In the experiments, the mechanical claw holes maintained the same sizes. The repair effects for the point cloud models are shown in Figure 15, and the chamfer distance between the repaired point cloud and the ground truth was calculated and is listed in Table 2. From the experimental results, it can be seen that our algorithm achieved a good hole repair effect for the point cloud models with different sizes, but the repair accuracy was slightly reduced when the mechanical claw holes occupied a large proportion of the whole point cloud model. Point cloud models with other shapes were also simulated, and the same conclusions were obtained.

#### 3.1.3. Comparative Experiment in Different Grasping Ways

In the real application scene, due to the different grasping angles and grasping positions of the mechanical arm, as well as the different placement directions of objects, the holes in the point cloud model appear in different spatial positions and have different shapes. In this section, the effectiveness of our algorithm is investigated for point cloud models of objects grasped in different ways. Cuboid point cloud models using different grasping ways were simulated as examples.

**Figure 16:**
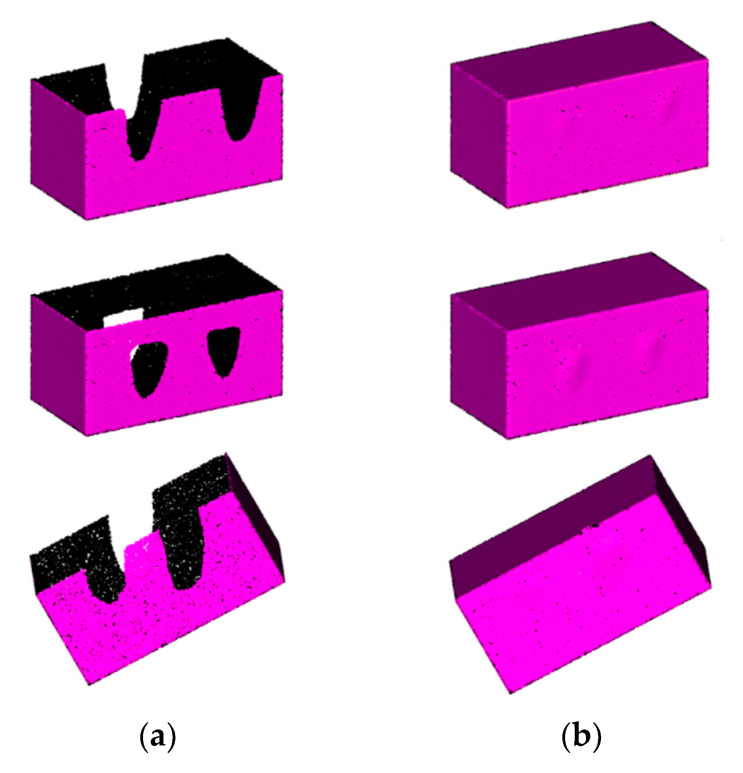
Repair results of cuboid point cloud models using different grasping ways: (**a**) before repair; (**b**) after repair.

The repair effect is shown in Figure 16; the chamfer distance was calculated and is listed in Table 3. From the experimental results, it can be seen that our algorithm has a robust hole repair effect for the object point cloud using different grasping ways.

### 3.2. Evaluation of Hole Repair Effect for Real 3D Point Cloud Model

**Figure 17:**
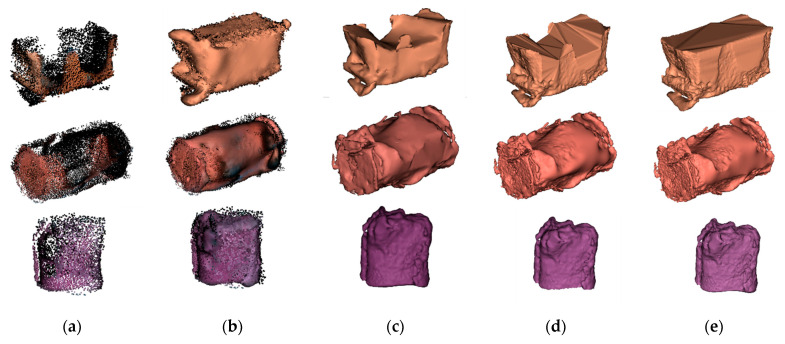
Repair effects of real point cloud models: (**a**) before repair; (**b**) ours; (**c**) DC-AB scanner; (**d**) Meshmixer; (**e**) Geomagic.

For the real point cloud of symmetrical objects grasped by the manipulator, the hole repair effects are shown in Figure 17. The hole-filling algorithms used by the DiChuang DC-AB 3D scanner [23], the Meshmixer software of Autodesk company [24] and the Geomagic Studio software of Geomagic company [25] were also adopted for comparison. The missing surface could not be repaired correctly by the DC-AB scanner or the Meshmixer software. Additionally, the repaired mechanical claw holes were not smooth enough. The hole repair effect with the Geomagic Studio software on the cuboid point cloud was slightly better than that of the other methods, but the whole side of the cylinder point cloud could not be repaired effectively. It can be seen that our algorithm achieved the best hole repair effects on the real point cloud models and could recover their shapes without destroying the original point cloud structure.

Compared with the simulated models in Section 3.1, our algorithm is not perfect for the real model. The main reason for this is that the point cloud is easily affected by the light, shadow and background in the real-time 3D reconstruction, which inevitably causes noise in the point cloud model. Additionally, it is also very difficult to remove the mechanical claws perfectly from the point cloud, which will affect the surface reconstruction to some extent.

## 4. Conclusions and Discussion

In this paper, the automatic hole repair algorithm is proposed for the 3D point cloud model of symmetrical objects grasped by the manipulator. Two kinds of holes, namely, the holes caused by the occlusion of the mechanical arm and the missing surface produced by the limited imaging angle, were automatically detected and classified. Then, they were repaired with surface reconstruction and symmetry completion. Experiments were performed on the simulated 3D point cloud models and the real acquired point cloud models. Compared with the hole-filling algorithms used by the DC-AB 3D scanner, the Meshmixer software and the Geomagic Studio software, the proposed algorithm achieved the best repair effects. Results also confirm its robustness in variable object sizes and different grasping ways of the manipulator. During the repair of the missing surface of the 3D point cloud model, the symmetry completion prior of objects is adopted. In the future, we will further relax this constraint and investigate the hole repair algorithm for asymmetric objects. Additionally, we will investigate and improve the hole repair accuracy when the holes occupy a large proportion of the whole point cloud model, which is still a very challenging task at present.

## Figures and Tables

**Table 1:** The chamfer distance between each simulated model and ground truth before and after repair.

Shape of Simulated Model	Before Repair	Ours	DC-AB Scanner	Meshmixer	Geomagic
Cuboid	33.81	**0.045**	5.72	2.28	0.25
Triangular prism	8.60	0.17	0.15	0.11	**0.10**
Cylinder	14.73	**0.14**	0.36	2.84	1.57
Sphere	6.40	**0.026**	1.08	0.071	0.088
Elliptic cylinder	37.76	**0.23**	41.50	40.57	0.70
Ellipsoid	9.86	**0.12**	4.46	0.20	0.21

**Table 2:** The chamfer distance for cuboid point cloud models with different sizes before and after repair.

Size of Cuboid Model (Unit: cm)	Before Repair	Ours
10 × 10 × 20	33.81	**0.045**
7 × 7 × 14	35.61	**0.049**
5 × 5 × 10	40.59	**0.15**

**Table 3:** The chamfer distance for cuboid point cloud models with different sizes using different grasping ways before and after repair.

Grasping Way	Before Repair	Ours
Top, Straight	35.61	**0.049**
Middle, Straight	32.76	**0.057**
Top, Oblique	35.89	**0.071**

## References

[1] Whelan T., Leutenegger S., Salas-Moreno† R.F., Glocker B., Davison A. ElasticFusion: Dense SLAM without a Pose Graph. Proceedings of the Robotics: Science & Systems.

[2] Chun-Yen C., Kuo-Young C. (2008). A sharpness-dependent filter for recovering sharp features in repaired 3d mesh models. IEEE Trans. Vis. Comput. Graph..

[3] Wei Z., Zhong Y., Yuan C., Li R. (2008). Research on smooth filling algorithm of large holes in triangular mesh model. China Mech. Eng..

[4] Wang Y.H., Wei-Yong W.U. (2009). Holes repairing algorithm of scattered data based on the fitting of partial subdivision curved surface. J. Mach. Des..

[5] Yang L., Yan Q., Xiao C. (2017). Shape-controllable geometry completion for point cloud models. J. Comput. Graph..

[6] Lin H., Wei W. Feature preserving holes filling of scattered point cloud based on tensor voting. Proceedings of the IEEE International Conference on Signal & Image Processing.

[7] Wang Y., Tang J., Zhao Y., Hao W., Lv K. Point Cloud Hole Filling Based on Feature Lines Extraction. Proceedings of the 2017 International Conference on Virtual Reality and Visualization (ICVRV).

[8] Tchapmi L.P., Kosaraju V., Rezatofighi H., Reid I., Savarese S. TopNet: Structural Point Cloud Decoder. Proceedings of the 2019 IEEE/CVF Conference on Computer Vision and Pattern Recognition (CVPR).

[9] Huang Z., Yu Y., Xu J., Ni F., Le X. PF-Net: Point Fractal Network for 3D Point Cloud Completion. Proceedings of the 2020 IEEE/CVF Conference on Computer Vision and Pattern Recognition (CVPR).

[10] Chang A.X., Funkhouser T., Guibas L., Hanrahan P., Huang Q., Li Z., Savarese S., Savva M., Song S., Su H. (2015). ShapeNet: An information-rich 3d model repository. Comput. Sci..

[11] ShapeNet Official Website. https://shapenet.org/.

[12] Sun D., Fan Z., Li Y. (2008). Automatic extraction of boundary characteristic from scatter data. J. Huazhong Univ. Sci. Technol..

[13] Zou D., Pang M. (2011). Algorithm for extracting sharp features from point cloud models. Trans. Chin. Soc. Agric. Mach..

[14] Removing Outliers Using a StatisticalOutlierRemoval Filter. https://pcl.readthedocs.io/projects/tutorials/en/latest/statistical_outlier.html#statistical-outlier-removal.

[15] Gu Y., Jiang X., Zhang L. (2008). Research and implement of hole-repairing technology for scatted point cloud. J. SooChow Univ..

[16] Fischler M.A., Bolles R.C. (1987). Random sample consensus: A paradigm for model fitting with applications to image analysis and automated cartography—Sciencedirect. Commun. ACM.

[17] Schnabel R., Wahl R., Klein R. (2010). Efficient ransac for point-cloud shape detection. Comput. Graph. Forum.

[18] Kazhdan M., Bolitho M., Hoppe H. Poisson surface reconstruction. Proceedings of the Fourth Eurographics Symposium on Geometry Processing.

[19] Kazhdan M., Hoppe H. (2013). Screened Poisson surface reconstruction. ACM Trans. Graph. (ToG).

[20] Lorensen W.E., Cline H.E. (1987). Marching cubes: A high resolution 3D surface construction algorithm. ACM Siggraph Comput. Graph..

[21] Newman T.S., Yi H. (2006). A survey of the marching cubes algorithm. Comput. Graph..

[22] Point Cloud Library (PCL)—pcl: Poisson< PointNT > Class Template Reference. https://pointclouds.org/documentation/classpcl_1_1_poisson.html.

[23] DISWAY, DC-AB 3D Scanner with High-Precision and Multipurpose. http://www.hbdc3d.com/smy/91.html.

[24] Autodesk Meshmixer—Free Software for Making Awesome Stuff. https://www.meshmixer.com/.

[25] Geomagic Releases Geomagic Studio 2012. https://www.3dsystems.com/press-releases/geomagic/releases-studio-2012?utm_source=geomagic.com&utm_medium=301.

